# 

**Published:** 1947

**Authors:** 


### Bristol Medico-Chirurgical Society

PROGRAMME FOR THE 1947–48 SESSION

December 10th.—Clinical Meeting at the Royal Fort.

January.—Dr. Campbell: "**Nervous Diseases in Animal and Man.**"

February.—Dr. Thompson, of Belfast, to read "**Malignant Disease of the Lung.**"

### The Medical Library of the University of Bristol

WITH WHICH IS INCORPORATED

**The Library of the Bristol Medico-Chirurgical Society**

*The following donations have been received since the publication of the list in the last issue.*

| | |
|---|---|
| Army Medical Library, Washington (1) | 1 volume. |
| Association of American Physicians (2) | 6 volumes. |
| Dr. W. P. Brinckman (3) | 2 volumes. |
| British Medical Association (4) | 1 volume. |
| Brompton Hospital (5) | 1 volume. |
| French Government (6) | 2 volumes. |
| Mr. G. C. K. Herapath (7) | 5 volumes. |
| Mrs. J. T. S. Hutchins (8) | 1 volume. |
| Dr. N. G. B. McLetchie (9) | 14 volumes. |
| Mayo Clinic (10) | 1 volume. |
| Michell Clarke Fund (11) | 1 volume. |
| Mrs. Norman Price (12) | 50 volumes. |
| J. E. Shaw Bequest (13) | 3 volumes. |
| University of Otago Medical School (14) | 1 volume. |
| Mrs. Wace (15) | 6 volumes. |
| Messrs. John Wright & Sons Ltd. (16) | 1 volume. |
| Yale University (17) | 1 volume. |

Unbound periodicals have also been received from the British Medical Association, Professor T. F. Hewer, Professor R. Milnes Walker and Messrs. John Wright & Sons Ltd.

### THE ONE HUNDRED AND EIGHTY-FIFTH LIST OF BOOKS.

The figures in round brackets refer to the figures after the names of the donors. The books to which no such figures have been attached have either been bought from the Library Fund or received through the *Journal.*

**Allardyce, M. D.** .. .. *The Library of the Medico-Chirurgical Society of Aberdeen* .. .. .. .. .. .. .. .. .. 1934

*Ane breif Description of the Well of Woman Hill besyde Abirdene* 1580. Facsimile. 1884

**Balfour, Sir A.** .. .. *Memoranda on medical diseases in tropical and sub-tropical areas* .. .. .. .. .. .. .. 8th Ed. 1946

**Beaumont, G. E. and Dodds, E. C.** *Recent advances in Medicine* 12th Ed. 1947

**Brain, W. R.** .. .. *Diseases of the Nervous System* .. .. 3rd Ed. 1947

**Brain, W. R. and Strauss, E. B.** *Recent Advances in Neurology* 5th Ed. 1946

**Burn, J. L.** .. .. .. *Recent Advances in Public Health* .. .. .. .. 1947

**Cameron, A. T.** .. .. *Recent Advances in Endocrinology* .. .. 6th Ed. 1947

*Care and Treatment of the Elderly and Infirm* .. .. .. .. .. .. .. (4) 1947

**Carling, Sir E. R. and Ross, J. P. (Ed.).** *British Surgical Practice* Vol. 1 (13) 1947

**Craig, W. S.** .. .. *Child and Adolescent Life in Health and Disease* (8) 1946

**Cushny, A. R.** .. .. *Pharmacology and Therapeutics* .. .. 13th Ed. 1947

**Dunlop, D.** .. .. .. *The Philosophy of the Bath* .. .. .. 4th Ed. 1880

**Edwards, C. T.** .. .. *Medicinal Properties of Bladud Spa Water* .. .. 1837

**Field, E. J. and Harrison, R. J.** *Anatomical Terms* .. .. .. .. .. .. 1947

**Fulton, J. F.** .. .. .. *Harvey Cushing* .. .. .. .. .. .. .. .. 1946

**Gregg, A.** .. .. .. *The Furtherance of Medical Research* .. .. (17) 1945

**Hadfield, G. and Garrod, L. P.** *Recent Advances in Pathology* .. 5th Ed. 1947

*Handbook of Preventive Medicine* (Air Ministry) .. .. .. .. .. .. .. 1947

**Harries, E. H. R. and Mitman, M.** *Clinical Practice in Infectious Diseases* 3rd Ed. (11) 1947

**Heffter, A.** .. .. .. *Handbuch der experimentellen Pharmakologie Erganzungswerk* .. .. .. Bde. 8, 9. 1939–41

**Hoffman, J.** .. .. .. *Female endocrinology* .. .. .. .. .. .. .. 1946

**Jameson, W. W. and Parkinson, G. S.** *Synopsis of Hygiene.* .. .. 9th Ed. 1947

**Johnson, A.** .. .. .. *Account of some Societies at Amsterdam and Hamburgh* .. .. .. .. .. .. .. .. .. 1773

**McMenemy, W. H.** .. *History of Worcester Royal Infirmary* .. .. .. 1947

**Mercer, W.** .. .. .. *Orthopaedic Surgery* .. .. .. .. .. 3rd Ed. 1947

**Moniz, E.** .. .. .. *Tentatives opératoires dans le Traitement de certaines Psychoses* .. .. .. .. .. (16) 1936

**Monrad-Krohn, G. H.** .. *Clinical Examination of the Nervous System* 8th Ed. 1947

**Peel, A. A. F.** .. .. *Diseases of the Heart and Circulation* .. .. .. 1947

**Perry, C.** .. .. .. *Account of an Analysis made on the Stratford Mineral Water* .. .. .. .. .. .. .. .. 1744

**Pye, W.** .. .. .. .. *Surgical Handicraft* .. .. .. .. .. 15th Ed. 1947

**Pyke, M.** .. .. .. *Manual of Nutrition* .. .. .. .. 2nd Ed. 1947

**Roxburgh A. C.** .. .. *Common Skin Diseases* .. .. .. .. 8th Ed. 1947

**Scott, J. A.** .. .. .. *National Health Service Act,* 1946 .. .. .. .. 1947

**Selye, H.** .. .. .. *Ovarian Tumors* 2 vols. .. .. .. .. .. .. 1946

**Slater, P.** .. .. .. *Survey of Sickness, Oct.* 1943 *to Dec.* 1945 .. .. 1946

**Smith, C. A.** .. .. .. *Physiology of the Newborn Infant* .. .. .. .. 1946

**Tidy, H. L. (Ed.)** .. .. *Inter-allied Conferences on War Medicine* 1942–1945 1947

| | | | |
|---|---|---|---|
| **Tidy, N. M.** .. .. .. | *Massage and Remedial Exercises* .. .. | 7th Ed. | 1947 |
| **Trueta, J. and others** .. | *Studies of the Renal Circulation* .. .. .. .. .. | | 1947 |
| **Whitby, Sir L.** .. .. | *Medical Bacteriology* .. .. .. .. .. | 4th Ed. | 1947 |
| **Wickersheimer, E.** .. | *Dictionnaire Biographique des Médecins en France au Nioyen Age.* 2 vols. .. .. .. .. .. | (6) | 1936 |
| **Woodworth, R. S.** .. | *Psychology* .. .. .. .. .. .. .. .. | 18th Ed. | 1946 |

TRANSACTIONS, REPORTS, JOURNALS, ETC.

| | | |
|---|---|---|
| Brompton Hospital Reports. Vol. 15 .. .. .. .. .. .. .. .. .. | (5) | 1946 |
| Collected Papers of the Mayo Clinic. Vol. 38, 1946 .. .. .. .. .. .. | (10) | 1947 |
| Journal of Laryngology and Otology Index. Vols. 46–55 (1931–1940) .. | | 1946 |
| Medical Directory, 1947 .. .. .. .. .. .. .. .. .. .. .. .. .. | | 1947 |
| Modern Treatment Yearbook, 1947 .. .. .. .. .. .. .. .. .. .. | | 1947 |
| Proceedings of University of Otago Medical School No. 24 .. .. .. | (14) | 1947 |
| Report on Ninth International Congress of Military Medicine and Pharmacy | (1) | 1937 |
| Transactions of the Association of American Physicians. Vols. 55–59. Index vols. 30–56. | (2) | 1940–47 |
| Transactions of Royal Humane Society, 1774–1784 .. .. .. .. .. .. | | 1795 |
| Yearbook of Dermatology and Syphilology, 1946 .. .. .. .. .. .. .. | | 1946 |
| Yearbook of Endocrinology, Metabolism and Nutrition, 1946 .. .. .. | | 1946 |
| Yearbook of Neurology, Psychiatry and Neurosurgery, 1946 .. .. .. | | 1946 |

### Publications Received

---

From JOHN WRIGHT & SONS LTD.:

*British Journal of Surgery.* Vol. 34. No. 136, 1947. Vol. 35. No. 137. Price 12s. 6d. Annual Subscription 42s.

*An Introduction to Gastro-enterology.* By JAMES D. HICKLEY, M.D. 1947. Price 8s. 6d.

*British Journal of Surgery War Supplement No.* 1. 1947. Price 30s. (Free to Subscribers to the *Journal.*)

*Hey Groves's Synopsis of Surgery.* 13th Edition. By SIR CECIL P. G. WAKELEY, K.B.E., C.B. 1947. Price 25s.

*Symptoms and Signs in Clinical Medicine.* By E. NOBLE CHAMBERLAIN, M.D., M.Sc., F.R.C.P. 4th Edition. 1947. Price 30s.

*Health Services in England.* By R. C. WOFINDEN, M.D., B.S., D.P.H., D.P.A. 1947. Price 10s.

From OXFORD UNIVERSITY PRESS:

*Diseases of the Nervous System.* By W. RUSSELL BRAIN, D.M., F.R.C.P. 3rd Edition. 1947. Price 37s. 6d.

From OXFORD UNIVERSITY PRESS (GEOFFREY CUMBERLEGE):

*Obstetrics and Gynaecology: A Revision Course for Practitioners.* By C. SCOTT RUSSELL, M.A., F.R.C.S., M.R.C.O.G. 1947. Price 12s. 6d.

From MESSRS. H. K. LEWIS & CO. LTD.:

*Common Skin Diseases.* By A. C. ROXBURGH, M.A., M.D., F.R.C.P. 8th Edition. 1947. Price 21s.

*Materia Medica for Nurses.* By A. MUIR CRAWFORD, M.D., F.R.F.P.S.G. 6th Edition. 1947. Price 5s. 6d.

From WM. HEINEMANN MEDICAL BOOKS LTD.:

*Diseases of the Joints and Rheumatism.* By KENNETH STONE, D.M., F.R.C.P. 1947. Price 30s.

*Cancer of the Breast.* By DUNCAN C. L. FITZWILLIAMS, C.M.G., M.D., F.R.C.S. 1947. Price 25s.

From HODDER AND STOUGHTON LTD.:

*Surgery: A Textbook for Students.* By C. A. PANNETT, B.Sc., M.D., F.R.C.S. 2nd Edition. 1947. Price 27s. 6d.

### Local Medical Notes

UNIVERSITY OF BRISTOL.

The following appointments have been conferred on former students and members of the staff :—

G. H. Bell, Professor of Physiology, University of St. Andrews.

T. B. Davie, Vice-Chancellor, University of Cape Town.

R. C. M. Hadden, Radiotherapist, Royal Devon and Exeter Hospital.

Duncan Wood, Director, Joint Cancer Committee for Devon and Cornwall.

J. M. Yoffey, Vice-President, Anatomical Society of Great Britain and Ireland.

M. Critchley, President, Harveian Society.

Veronica Dawkins, Physician, British Legion Sanatorium, Nayland.

W. S. Ormiston, A.D.M.S., Nigeria.

B. H. Page, Surgeon, North Middlesex County Hospital.

The following appointments have been made : A. Rendle Short, *Emeritus Professor of Surgery.* D. H. Davies, A. M. G. Campbell, *Lecturers in Medicine.* H. Caplin, *Lecturer in Pathology.* P. Jardine, *Clinical Teacher in Ophthalmology.* R. G. Paul, *Lecturer in Operative Surgery.* J. V. Sparks, *Lecturer in Radiodiagnosis.*

SCHOLARSHIPS AND PRIZES.

*Beaverbrook Fellowship.*—H. D. Moore.

*Sidney Robinson Fellowship.*—M. H. L. Desmarais.

*Royal Hospital Board's Gold Medal.*—K. D. J. Vowles.

*Royal Hospital Board's Silver Medal.*—C. D. R. Pengelly.

*Martyn Memorial Scholarship.*—P. W. Seargeant.

*Open Entrance Scholarships.*—W. E. Benney, Ruth J. Grose.

*Edward Fawcett Memorial Prize.*—R. J. Ancill.

*Thomas Francis Edgeworth and Francis Henry Edgeworth Prize.*—R. J. Ancill.

*Foyle Prize.*—R. J. Ancill.

*Mackie Prize.*—W. J. Gall.

*Dental Gold Medal.*—P. J. Sheldrick.

*De Trey Prize.*—R. J. Brownlee.

EXAMINATION RESULTS.

**University of Bristol.**—Members of the University have passed the following examinations :—

M.B., CH.B.—*Second Examination* (*Section II*) *:* Margaret V. Brown, M. A. Feldman, G. A. K. M'C. Jahans, K. R. Kidd, S. J. Lloyd, J. H. Scudamore, R. E. D. Simpson, Patricia Vowles.

**Royal Colleges—Conjoint Board.**—M.R.C.S., L.R.C.P.—N. M. Gibbs (1), D. M. Cunningham (1), E. M. R. Davies (2), G. Winternitz (2), R. J. Carey (3).

BRISTOL ROYAL HOSPITAL.

The following appointments have been made : C. D. Evans, *Honorary Dermatologist.* G. L. Feneley, *Honorary Anaesthetist.* G. D. Kersley, *Honorary Physician for Chronic Rheumatic Diseases.* A. V. Neale, *Honorary Physician.* J. P. Raban, *Radiotherapeutist.*

UNIVERSITY OF BRISTOL DENTAL HOSPITAL.

G. F. Fawn has been appointed *Honorary Consulting Dental Surgeon.*

### CHRISTMAS, 1947.

The Library will close at 5 p.m. on December 23rd and will re-open at 9 a.m. on January 1st, 1948.

